# An Optically Stabilized Fast-Switching Light Emitting Diode as a Light Source for Functional Neuroimaging

**DOI:** 10.1371/journal.pone.0029822

**Published:** 2012-01-06

**Authors:** Daniel A. Wagenaar

**Affiliations:** Broad Fellows Program and Division of Biology, California Institute of Technology, Pasadena, California, United States of America; Freie Universitaet Berlin, Germany

## Abstract

Neuroscience research increasingly relies on optical methods for evoking neuronal activity as well as for measuring it, making bright and stable light sources critical building blocks of modern experimental setups. This paper presents a method to control the brightness of a high-power light emitting diode (LED) light source to an unprecedented level of stability. By continuously monitoring the actual light output of the LED with a photodiode and feeding the result back to the LED's driver by way of a proportional-integral controller, drift was reduced to as little as 0.007% per hour over a 12-h period, and short-term fluctuations to 0.005% root-mean-square over 10 seconds. The LED can be switched on and off completely within 100 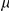
s, a feature that is crucial when visual stimuli and light for optical recording need to be interleaved to obtain artifact-free recordings. The utility of the system is demonstrated by recording visual responses in the central nervous system of the medicinal leech *Hirudo verbana* using voltage-sensitive dyes.

## Introduction

Neuroscientists more and more frequently turn to optical methods for both stimulation and recording of neuronal activity (e.g., [1], [2]). To attain optimal results from experiments using voltage-sensitive or calcium-sensitive dyes, brightness and stability of the excitation light source are critical, as fluorescence signals are often small [3], [4]. The ability to switch excitation light rapidly on and off is often important as well, especially when light is also used for stimulation (either in the form of visual stimuli or of direct optical stimulation of neurons). This often requires interleaving stimuli and functional imaging at a time scale of milliseconds: While optical filters can separate the wavelengths in favorable circumstances, the fluorescence changes produced by physiological changes in membrane potential or calcium concentration commonly are so small (or the wavelength separation so narrow) that optical filters cannot sufficiently suppress the variable background induced by the stimulation light. Thus image acquisition must be disabled during stimulation, and because many voltage and calcium dyes are phototoxic, it is highly undesirable to leave the excitation light on when image acquisition is disabled; hence the need for fast switching.

For decades halogen and arc lamps have been the light sources of choice for microscopy [5]. Of the two conventional technologies, arc lamps were by far the brightest, which made them the obvious choice for fluorescence microscopy. However, a shortcoming of arc lamps has always been their stability: for precision experiments with voltage-sensitive or calcium-sensitive dyes, their flickering and drift is often unacceptable. This is especially true for mercury arc lamps, but even so-called “super-quiet” xenon arc lamps are not as quiet as a well-stabilized halogen lamp [6], which can be extremely stable when used with a high-quality power supply. However, halogen lamps are not as bright as arc lamps. Both types of lamp take seconds (or more) to switch on or off. As a consequence of these limitations, these conventional technologies are more and more commonly replaced by lasers and light-emitting diodes (LEDs).

Lasers are the illumination source of choice for many types of modern microscopy, including confocal microscopy and multi-photon microscopy [7], and their use is expanding as prices come down. One disadvantage lasers share with halogen and arc lamps is that they need time to warm up to attain beam stability, and hence cannot be switched on and off rapidly. They are however unquestionably the brightest light sources available.

Although LEDs cannot pack as much light into a narrow beam as lasers do, they are an increasingly attractive alternative to arc or halogen lamps as brighter and more cost-effective devices become available every year. LEDs can be switched on and off rapidly. They consume less energy than conventional light sources and consequently can be controlled with simpler electronics and produce less heat. Most LEDs emit in a single, relatively narrow wavelength band which is convenient for fluorescence microscopy. When multiple wavelengths are required in an imaging experiment, the output of several LEDs can readily be combined with dichroic mirrors, or white LEDs can be considered.

One problem with LEDs is that their brightness and spectral properties are sensitive to temperature variations (e.g., [8]). Rather than trying to control these temperature fluctuations, this paper describes a system that uses feedback from a photodiode to stabilize LED output. Analogous systems have been used to stabilize lasers [9] and mercury arc lamps [10]. Commercial implementations of this concept for LED sources are beginning to appear (e.g., OptoLED, Cairn Research, Faversham UK, and LEX2, SciMedia, Costa Mesa CA), but their (limited) published specifications are not better than the best stability from a non–feedback controlled LED source reported in the literature [11]. Thanks to the optical feedback, the stability of the LED source described in this paper is on par or better than that of a halogen lamp at all time scales, while the brightness is on par with that of a mercury arc lamp. As an example to demonstrate the system's utility in a real-world situation, voltage-sensitive dye recordings were obtained from live neurons under conditions where conventional light sources are inadequate.

## Methods

**Figure 1:**
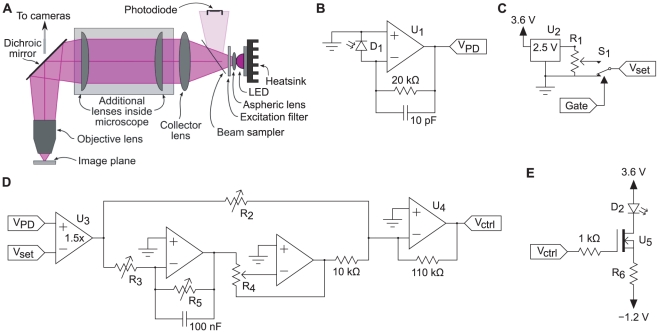
Design of the LED light source. **A.** Overview of the optics. **B.** Photodiode circuit. **C.** Reference voltage circuit. **D.** Proportional-integral controller. **E.** LED power circuit. For details, see text.

A high-power (500 mW light output) LED with emission at 405 nm (LZ1-10UA05-U7, LedEngin, Santa Clara CA) was mounted on a heat sink mounted on a filter changer (Thor Labs, Newton NJ) mounted on an x-y-z translation stage (Siskiyou, Grants Pass OR). An optical bandpass filter (D405/30x, Chroma, Bellows Falls VT) was glued in front of the LED. A biconvex lens (f
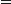
85 mm, diameter: 50 mm; Anchor Optics, Barrington NJ) was positioned in the place of a halogen light house on the fluorescence input of an upright microscope (Examiner A1, Zeiss, Jena, Germany). The LED was moved until the microscope projected its image in the same plane and x-y location where it normally projects the image of the halogen lamp, i.e., approximately 5 cm below the flange of the objective lenses. A beam sampler (Thor Labs BSF10-A) was placed at a 45 angle in front of the LED, and a photodiode (S1226-8BK, Hamamatsu, Bridgewater NJ; D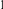 in Figure 1B) was mounted so as to sample the light going to the microscope. The entire setup is depicted in Figure 1A.

The photocurrent from the photodiode was converted to a voltage using a zero-drift opamp (LTC1152, Linear Technology, Milpitas CA; U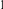 in Figure 1B). A 10-pF capacitor ensures stability without compromising response times. (The time constant of the circuitry around U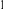 is 200 ns, considerably faster than the response time of the LED).

The output voltage from U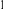 was compared to a set point based on an ultrastable 2.5 V reference (Linear Technology LT1019; U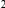 in Figure 1C). The set point voltage was routed through an electronic switch (MAX4619, Maxim, Sunnyvale CA; S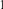) to enable fast switching between the set point and no light. The comparison itself was performed by a precision instrumental amplifier (INA118, Texas Instruments, Dallas TX; U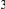 in Figure 1D) and a simple proportional-integral controller (Figure 1D). The memory duration of the integral branch could be varied between 0 and 100 ms by tuning R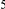 (1 M 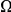).

The output of the controller was used to drive the current through the LED (D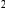 in Figure 1E) by way of an n-channel power FET (FQP30N06L, Fairchild Semiconductor, San Jose CA; U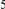). Stability was improved by the series resistor R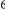 (0.47 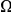). Limiting the bandwidth of the output stage U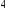 (LTC1152) by way of a small capacitor in parallel with the feedback resistor did not prove beneficial.

The complete circuit diagram, which includes additional circuitry to discharge the integrator as well as a safety cutoff switch to prevent runaway LED currents in case of photodiode dislocation, is available on request. These additions did not significantly affect normal function and were omitted from Figure 1 for clarity. The entire circuit was powered by 4 AA-sized NiMH rechargeable batteries which provided a positive supply of 3.6 V and a negative supply of 1.2 V.

All measurements of light output and noise levels were made with an external photodiode (PC2-2-TO18, Pacific Silicon Sensor, Westlake Village CA) connected to an amplifier identical to the one used in the feedback circuit (Figure 1B) and placed in the focal plane of a microscope. Data were recorded using a digital storage oscilloscope (TDS 2004B, Tektronix, Beaverton OR) or using a computer-based data acquisition system (USB-6029, National Instruments, Austin TX) and analyzed in Matlab (Mathworks, Natick MA).

For tuning the circuit, 100-ms long TTL pulses were applied to the gate input of the circuit, and the actual light output as measured by the external photodiode was monitored on a digital oscilloscope. The gain of the integral branch of the controller was first set to a low value using variable resistors R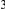 and R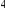 (both 100 k 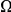, Figure 1D), and the gain of the proportional branch was set to its minimum using variable resistor R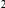 (100 k 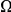). The memory time constant of the integral branch was then tuned to about 10 ms using R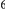 (1 M 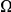) and the gain of the integral branch was increased until overshoot became apparent, then slightly reduced from that point. Finally, the gain of the proportional branch was increased to improve response times.

**Figure 2:**
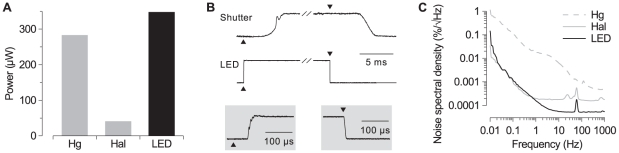
Feature comparison of three different light sources for fluorescence microscopy. **A.** Brightness: optical power in an 8-nm wide wavelength band centered at 405 nm. Hg: mercury arc; Hal: halogen; LED: custom LED. **B.** Time course of photodiode current after switching illuminators on (upward triangles) and off (downward triangles): mechanical shutter and electronic LED circuit. Inset: detail of LED timing at shorter time scale. **C.** Noise spectra of light output as measured by a photodiode. Hg, Hal, LED as in (A).

**Figure 3:**
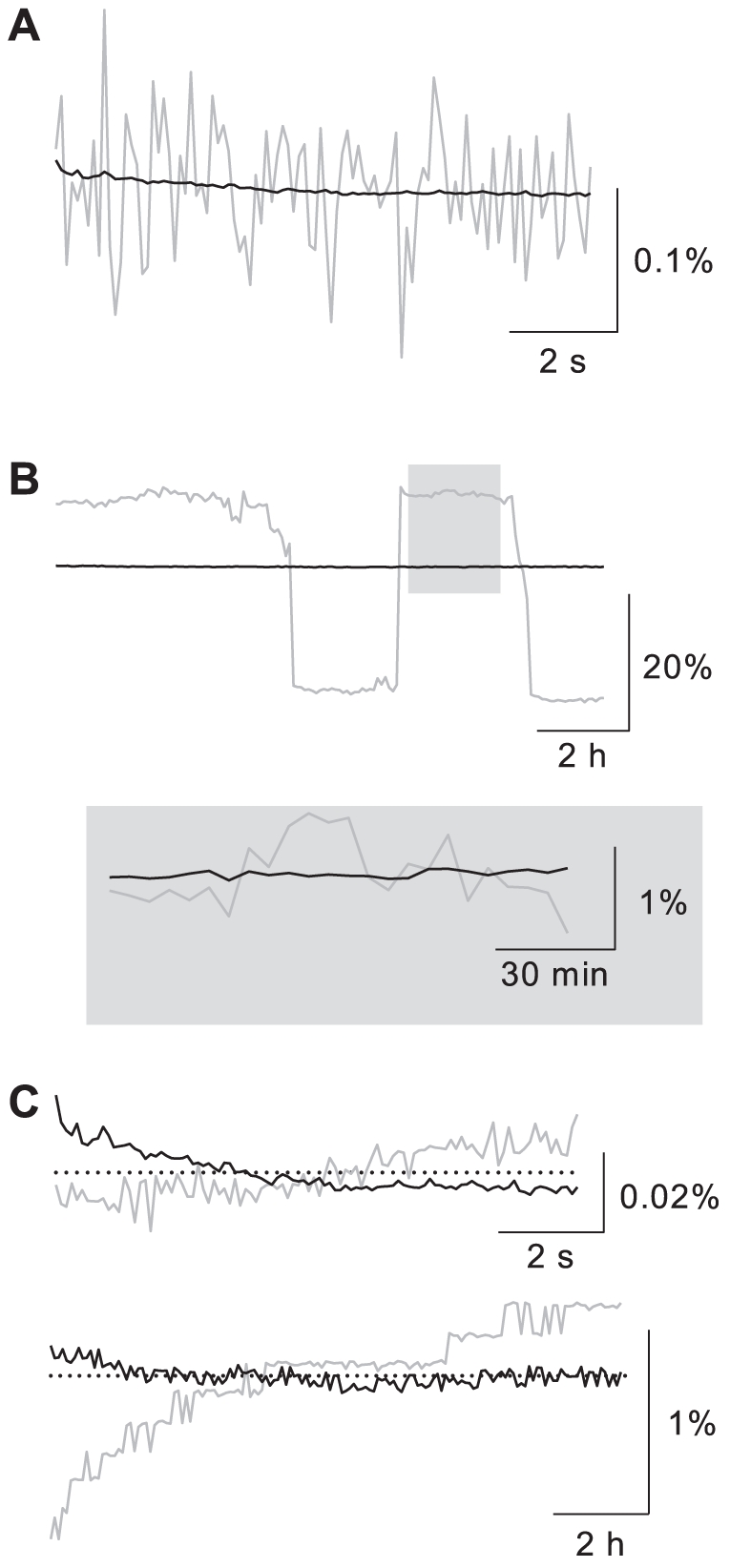
Comparison of LED source with mercury arc lamp at two time scales of particular interest. **A.** Fluctuations in the output of the LED source (black) and the mercury arc lamp (gray) in a 10-s long sequence of 35-ms exposures at 10 Hz. **B.** Fluctuations in the outputs of the same sources in a 12-h long sequence of 500-ms exposures at 5 minute intervals. Inset: detail at higher magnification. **C.** Drift and noise of measured light output from LED lamp with (black) and without (gray) optical feedback at short time scale (top) and long time scale (bottom). Please see text for comparison to a current-generation xenon arc lamp.

To achieve the highest possible stability, placement of the feedback photodiode was critical. If the feedback diode was moved to face the LED directly but at high angular eccentricity (rather than through a beam sampler as shown in Figure 1), light levels measured at different positions in the optical path of the microscope could vary by more than 0.1% even when the output of the feedback diode was flat within 0.01%. Temporarily blocking the light to the test diode while leaving the LED on did not produce a new transient in the test diode's output, ruling out that thermal effects in the test diode were responsible. The amount of light power hitting the feedback diode was no greater than that hitting the test diode, making it unlikely that thermal effects in the feedback diode were responsible either. Since the size (and sign) of the transients depended on the eccentricity of the feedback diode relative to the optical axis (data not shown), it appears likely that temperature changes affected the shape of the LED's internal lens, which would in turn affect the shape of the output beam. The use of a beam sampler to direct light from the central part of the LED's output to the feedback diode reduced these effects greatly. Similarly, the use of a single optical filter rather than one excitation filter in the microscope's turret and a separate one in front of the feedback photodiode reduced the differences in light levels registered by the test and feedback diodes. These two observations together were pivotal to attaining the level of stability demonstrated in Figures 2C and 3.

The performance of the LED source was compared with two alternative light sources in common use in our lab. One was a typical mercury arc lamp (Nikon Intensilight C-HGFI, 130 W), chosen as a target for brightness. This lamp was not specifically designed to be highly stable, but while more stable arc lamps are on the market (see Results), these were not available for this study. The other lamp used for comparison was a halogen lamp (EIKO FCR, 100 W) powered by an external power supply (JQE-15-12, KEPCO, Flushing NY) with error sensing on the drive voltage. In contrast to the arc lamp mentioned above, this combination of lamp and power supply was specifically chosen to optimize stability.

For biological experiments, medicinal leeches (*Hirudo verbana*; obtained from Niagara Leeches, www.leeches.biz) were maintained as described before [12]. Animal care was in accordance with best practice in the field; no institutional or NIH guidelines exist for lower invertebrates such as leeches. Leeches were anesthetized in ice-cold water and opened along the dorsal midline, and a portion of body wall consisting of three adjacent segments was excised and placed skin-down on a Sylgard-covered petri dish. Only the ganglion in the central segment was left connected to the periphery. The ventral surface of the ganglion was desheathed, and coumarin (N-(6-chloro-7-hydroxycoumarin-3-carbonyl)-imyristoylphosphatidylethanolamine; Vertex Pharmaceuticals, San Diego CA) and oxonol (bis (1,3-diethyl-thiobarbiturate)-trimethine oxonol; Vertex) were applied to it as described previously [13]. Coumarin and oxonol form a voltage-sensitive dye (VSD) pair that responds to membrane voltage changes with a fluorescence change of about 5% per 100 mV and a time constant of about 450 ms [14]: Coumarin is a large molecule that is bound to the outer leaflet of the cell membrane, while oxonol is a smaller molecule with positive net charge that can move inward and outward through the membrane. Depolarization of the cell therefore brings the two molecules closer together, resulting in increased Förster resonance energy transfer (FRET) [15]) from coumarin to oxonol, and hence a reduction of the fluorescent emission from coumarin and a corresponding increase of the emission from oxonol. Thus, changes in the ratio between the two emission channels can be used as a measure of membrane voltage changes.

**Figure 4:**
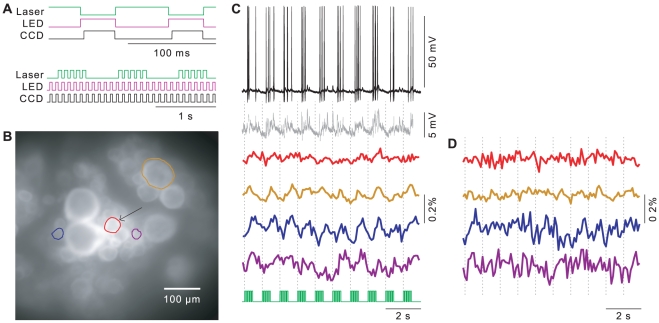
Real-world test of the LED light source. **A.** Timing diagram of visual stimuli delivered to peripheral sensilla (“Laser”), excitation light for voltage-sensitive dyes (“LED”) and frame acquisition by the two CCD cameras (“CCD”), at the short time scale of single frames (top) and the longer time scale of effective light stimuli (bottom). **B.** Fluorescence micrograph of a leech ganglion (detail) with several cells indicated. The arrow indicates a neuron known as the “S” cell. **C.** Intracellular recording (black) from the S cell, same data with action potentials digitally removed (gray), and simultaneously recorded ratiometric VSD fluorescence from the S cell (red) and the other cells circled in (B). Timing of the visual stimuli is indicated in green. **D.** VSD signals after addition of synthetic noise to simulate recording conditions with a halogen lamp.

A fast-switchable green laser (20 mW at 532 nm, Virtual Village, Hong Kong, China) and a pair of fast steering mirrors (20 kHz nominal rate, Optic Pic, selling through eBay) were used to project user-defined patterns of light onto the visual sensilla [16] in the body wall. A neutral density filter was used to limit the brightness of the stimuli to 2500 lux. Images were acquired at 10 Hz with a 35% duty cycle (i.e., 35 ms exposure time); of the remaining 65 ms frame period, 60 ms was used for visual stimulation, and 5 ms to provide a (generous) margin to avoid polluting the VSD image sequence with visual light (see Figure 4A). To minimize phototoxicity, the LED was on only during CCD exposure. Images of FRET donor and acceptor fluorescence were acquired using a pair of CCD cameras (QuantEM 512SC, Photometrics, Tucson AZ) controlled by custom software, and changes in the ratio of oxonol/coumarin fluorescence in hand-drawn regions of interest were calculated in single trials. The effects of photobleaching were digitally suppressed to second order. Simultaneously, the membrane potential of one selected neuron was recorded using an intracellular electrode (impedance: 30 M 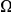; fill solution: 3 M potassium acetate, 20 mM potassium chloride). Intracellular signals were amplified using an Axoclamp-2A amplifier (Axon Instruments, now Molecular Devices, Sunnyvale CA) and digitized at 10 kHz (National Instruments USB-6029). Data acquisition was controlled by custom software.

## Results

An external photodiode (see Methods) was mounted in the focal plane of a microscope, and used to measure the light output of three illuminators:

- a mercury arc lamp (see methods) projecting through a 10×, 0.30 N.A. objective on an inverted fluorescence microscope (Nikon Eclipse TE2000-U);
- a halogen lamp (see methods) mounted in a Zeiss HAL 100 illuminator and projecting through a 10×, 0.25 N.A. objective on an upright fluorescence microscope (Zeiss Examiner A1); and
- our custom LED source on the same upright microscope with the same objective.

The first measurement concerned the overall light output of each source. For this measurement, all sources were turned to their maximum brightness and light was filtered through a narrow bandpass filter around 405 nm (Chroma D405/8x). In all cases, the microscope's focus was adjusted and the stage was translated in the x-y plane so as to maximize the photocurrent in the diode. Because different microscopes were used for the measurements, the results (Figure 2A) are somewhat qualitative. Even so, it is safe to conclude that the LED source outputs approximately as much energy as the mercury arc lamp in the wavelength band of interest, and much more than the halogen lamp. Figure 2B compares the switch speed of the LED source with an electromechanical shutter of a type (VS25, Uniblitz, Rochester NY) commonly used in conjunction with mercury arc or halogen lamps. (Note that it is possible in principle to project such lamps through a smaller and hence faster shutter, but this is not commonly done.) There was a 3-ms delay between the trigger signal and the time the shutter began opening, and it took another 3 ms for the shutter to fully open. For closing, the delay was 4 ms and closing itself took 3 ms. In contrast, the LED reached full brightness within 75 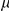
s after the trigger signal, and fully extinguished within 10 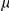
s.

An extremely important property of light sources for quantitative fluorescence microscopy is stability over a range of time scales. The continuous light output from all three light sources was measured in multiple 5 minute trials, and the noise was calculated as the deviation from baseline. The noise spectra are presented in Figure 2C; the wide-band noise (10 mHz to 10 Hz) was 0.095%
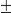
0.011%, 0.0035%
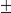
0.0012%, and 0.0022%
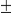
0.0010% root-mean-square (RMS) for the mercury arc, halogen, and LED sources, respectively (mean 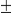 std. dev.; 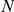 = 5). At high frequencies (above 10 Hz), the measurements from both the halogen lamp and the LED were shot-noise limited, and, naturally, the shot noise is larger for the dimmer halogen lamp. (The small peaks at 60 Hz are due to pickup of U.S. line noise.) It is important to note that all measurements were performed using an external photodiode, not with the photodiode that is part of the optical feedback circuit, as using the latter would unfairly bias the results in favor of the LED source.

Figure 3 compares the stability of the LED light source with the mercury arc lamp on two different time scales: the time scale of seconds, which is important for VSD imaging of synaptic dynamics [17], [18] or leech behavior [13], [19], and the time scale of hours, which is important for long-term recording of neural activity (e.g., [20]). For the short time scale, light output was measured in a 10-s long image sequence at 10 frames per second, with 35% duty cycle. The LED source exhibited fluctuations of 0.0052%
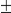
0.0002% RMS (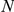 = 3), the mercury arc source 0.062%
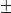
0.026% RMS (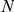 = 40) (Figure 3A).

For the long time scale, a 12-h long sequence was used that consisted of 500-ms long exposures every 5 minutes (Figure 3B). The RMS noise over the entire sequence was 0.05% for the LED and 13% for the mercury arc lamp. This latter figure was dominated by sharp transitions between two semistable states, but even disregarding any jumps of more than 1% between two consecutive exposures, the RMS noise of the mercury arc lamp was more than 30× greater than that of the LED: 1.7%. It should be noted that there are now arc lamps on the market with considerably lower noise than the mercury arc lamp used here. For instance, the Hamamatsu L11033 xenon arc lamp is specified to have fluctuations of 0.2% RMS.

Over this 12-h period, the temperature in the lab fluctuated by several degrees. Perhaps as a result, the photodiode recorded a long-term drift in the LED output of −0.007% per hour, although it is not certain that this drift was (entirely) in the LED output rather than in the photodiode amplifier. To rule out the possibility that the feedback circuit might actually add to drift on this time scale (which would be possible if the LED settles after a few hours of stationary operation), a second overnight recording was made for which the LED was powered by the KEPCO power supply using a constant-current configuration rather than optical feedback. This resulted in a tenfold higher drift: +0.08% per hour (Figure 3C), confirming that the feedback circuitry was highly beneficial at this time scale. At the very shortest time scale, the KEPCO could keep the LED almost as stable as the feedback circuit (0.0018% drift per second vs. −0.0015%) although it should be noted that this measurement was taken without any shuttering, so this result may be biased in favor of the KEPCO.

This new bright, stable, and fast-switchable light source opens up experimental possibilities that were not available with conventional light sources. One example of this is voltage-sensitive dye (VSD) recording of neuronal responses to visual stimuli in the medicinal leech. The best VSD currently available for use in this animal [14] has relatively low sensitivity which means that a bright light source with extremely low noise is required. Furthermore, phototoxicity requires that the light be on for as short a time as possible.

Green light was projected onto the light-sensitive sensilla [16] in a short section of the body wall of a medicinal leech, while recording from an attached ganglion using voltage-sensitive dyes and an intracellular electrode. Due to the small size of the animal, it was not practical to fully prevent stray light from the visual stimuli from entering the microscope objective, so stimulation of the sensilla was interleaved with VSD excitation and recording (Figure 4A, and Methods). Stimulation of the sensilla consisted of wide-field illumination at 1 Hz with a 50% duty cycle. Because of interleaving, each “on” phase of the cycle consisted of 5 pulses of 60 ms each, at 10 Hz; excitation of the VSDs and image acquisition of their fluorescence signals occurred in the interval between these pulses (Figure 4A).

From several neurons that have previously been found to respond to light flashes [16], I chose to focus on the S cell (red circle and black arrow in Figure 4B), a small interneuron in the medial packet of the ganglion [21]. This cell was chosen because it responds reliably to light, and because it is small, making for a challenging and therefore interesting test case for VSD imaging. Indeed, the S cell responded to light flashes (green trace in Figure 4C) with bursts of action potentials clearly visible in the intracellular recording of membrane potential (black trace). The simultaneously obtained VSD signal (red trace), though small in amplitude as is typical for the dyes used, followed the corresponding membrane depolarizations (coherence at 1 Hz: 0.86
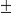
0.10). (Individual action potentials cannot be seen in the VSD trace, since the time constant of the dye is too slow).

A key reason to choose VSD imaging over intracellular recording is the promise of recording from many cells at once. Indeed, in the present experiment, several other cells responded at least as strongly to the visual stimuli as the S cell did. These cells are circled in Figure 4B and their VSD signals are shown in Figure 4C. Since both LED and halogen lamps were shot-noise limited above 1 Hz (Figure 2C), it was possible to simulate what these signals would have looked like had they been acquired with the halogen lamp instead. When synthetic shot noise was added to the acquired images based on the brightness difference of the two sources (Figure 2A), the stimulus-related oscillations in the VSD signals were buried in the noise (Figure 4D).

## Discussion

This paper introduces an LED-based light source (Figure 1) for quantitative fluorescence microscopy that, within its narrow wavelength band, is as bright as a mercury arc lamp (Figure 2A), yet has both short-term and long-term stability properties on par with the highest achievable standard with halogen lamps (Figures 2C and 3). This stability was achieved by regulating the current to the LED based on feedback from a photodiode. In comparison to a commercial product based on similar principles (SciMedia LEX2), the source described here achieved higher stability: 0.005% fluctuations vs 0.015% and 0.0015%/s drift vs. 0.025%/s. (LEX2 figures taken from company website.) The contrast with commercial laser sources is greater: current lasers from Coherent (Santa Clara CA) and Newport (Irvine CA) are specified to offer high-frequency noise below 0.05% RMS and long-term drift below 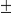
2% over 8 hours. A final worthwhile comparison is with current generation xenon arc lamps: these are offered with quoted fluctuations of 0.2% RMS and drift of 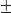
0.5%/hour (Hamamatsu), which puts them at an intermediate position between older arc lamps and the stabilized LED source.

Importantly, LEDs can be switched on and off with sub-millisecond speed (Figure 2B), allowing the excitation light to be kept off except during actual image acquisition, thus minimizing phototoxicity. In contrast, neither arc lamps nor halogen lamps can be switched on or off rapidly, so mechanical shutters are generally employed to regulate the illumination, but even high-quality shutters have response delays of the order of several milliseconds and then take another several milliseconds to fully open or close. As an added bonus, LEDs are very energy efficient, making it possible to operate the entire circuit using battery power.

The regulation used for this light source is similar in approach to the ones used by [9] and [10] for VSD imaging, but offers several improvements. First, the present design provides regulation all the way down to DC, allowing for stability on arbitrarily long time scales. Second, the regulator can switch the light source all the way off with sub-millisecond timing. Lastly, the present design benefits from all the other advantages of LED technology including low heat generation, low cost, and long life. In comparison to the excellent non–feedback controlled LED light source reported by [11], the source reported here is 2× more stable, and, importantly, this stability is reached immediately rather than after several seconds of warm-up time. The short-term drift shown in Figures 2A and 2C could be further reduced by adding a mechanical shutter to the optical path and starting the LED light pulse sequence several seconds before the light is used for imaging.

The utility of the described light source was demonstrated by using it to provide excitation light for voltage-sensitive dye recording from the nervous system of a leech in a semi-intact preparation that received simultaneous visible-light stimulation (Figure 4). Fluorescence signals were recorded in single trials that were so small that they would have been invisible if a halogen or mercury arc lamp had been used: With a halogen lamp, the excitation light would have been almost tenfold dimmer so that the signal would have been hidden in photon shot noise (Figure 4D), whereas the limited stability of mercury arc lamps would have produced at least tenfold higher baseline fluctuations, which would also have buried the signal (data not shown).

Naturally, the use of the described light source is not limited to this type of experiments or to the leech as a model system. While this paper presents results obtained with a 405-nm LED, the feedback system could equally well regulate the output of LEDs of any other wavelength, and, by mounting several LEDs on a filter wheel, rapid manual or motorized switching between excitation light of several wavelengths can be conveniently achieved. Further, when multiple wavelengths are required simultaneously, the output of multiple illuminators of the type described here can readily be combined with dichroic mirrors. Thus, it is straightforward to accommodate a wide variety of dyes and experimental preparations.
